# Factors contributing to the sustained implementation of an early childhood obesity prevention intervention: The *INFANT Program*

**DOI:** 10.3389/frhs.2022.1031628

**Published:** 2022-11-25

**Authors:** Penelope Love, Rachel Laws, Sarah Taki, Madeline West, Kylie D. Hesketh, Karen J. Campbell

**Affiliations:** ^1^Faculty of Health, School of Exercise and Nutrition Sciences (SENS), Institute for Physical Activity and Nutrition (IPAN), Deakin University, Geelong, VIC, Australia; ^2^School of Public Health, Faculty of Medicine and Health, University of Sydney, Camperdown, NSW, Australia; ^3^Health Promotion Unit, Population Health Research and Evaluation Hub, Sydney Local Health District, Sydney, NSW, Australia; ^4^Institute for Mental and Physical Health and Clinical Translation (IMPACT), Food and Mood Centre, School of Medicine, Deakin University, Geelong, VIC, Australia

**Keywords:** implementation, sustainability, maintenance, early childhood, obesity prevention, health promotion

## Abstract

**Background:**

The *INFANT Program* is an efficacious, group-based program for first-time parents, delivered at three-monthly intervals when *INFANT* are aged 3–18 months through an existing universal care service in Victoria, Australia. Many lessons have been learnt from its origins as a cluster randomized control trial to its small-scale, community-level implementation. This study aimed to describe factors contributing to its sustained implementation to inform large-scale implementation across Australia.

**Methods:**

This study used a multi-site qualitative exploratory approach. *INFANT* facilitators trained between 2013 and 2017 were sent an online survey, with optional telephone interviews. The Consolidated Framework for Implementation Research (CFIR) was selected as the underpinning theoretical framework as it offered the opportunity to explore a breadth of possible barriers and enablers across patterns of implementation (never, discontinued, ongoing).

**Results:**

All participants were female (*n* = 31), the majority were Maternal and Child Health Nurses (48%), representing five regional and nine metro local government areas (LGAs), across all patterns of implementation (never implemented *n* = 4; discontinued implementation *n* = 5; ongoing implementation *n* = 5). All consenting participants were interviewed (*n* = 11) representing four regional and seven metro LGAs, across all patterns of implementation (never implemented *n* = 3; discontinued implementation *n* = 4; ongoing implementation *n* = 4). The main reason for attending *INFANT Program* training was to become skilled to implement the program. Mapping identified barriers and enablers to the CFIR revealed the inner and outer settings and implementation process to be of greatest influence. Main differences between LGAs with ongoing and discontinued implementation related to funding availability, organizational management support and endorsement, organizational resourcing and capacity, integration into routine practice and establishing role clarity with partner organizations, and planning for sustained implementation from the start.

**Conclusion:**

This study provides important insights into the barriers and enablers to the sustained implementation of an evidence-based intervention (the *INFANT Program*) during small scale community-level implementation. The authors therefore contend that the pre-requisite for scale-up of a population health intervention is not just proof of effectiveness but also proof of sustained implementation at the local/organizational level. Study findings have broad transferability given their similarity to those identified for health promotion interventions implemented globally, in healthcare, education and community settings.

## Introduction

The first 1,000 days (conception to 24 months) are acknowledged as a crucial period for growth and development in early childhood, laying the foundation for life-long health behaviors and the prevention of chronic disease (1, 2). The early establishment of healthy behaviors (3), such as prolonged breastfeeding (4), reduced consumption of energy-dense, nutrient-poor foods/beverages (5), limited screen time and sedentary behavior (6), and prevention of rapid infant weight gain (7), is considered critical for the prevention of childhood obesity and overweight which affects an estimated 38.3 million children under the age of 5 years globally (8). In Australia, 25% of children aged 2–4 years are already experiencing overweight/obesity (9), with a minority meeting the recommended dietary and movement guidelines (10), and those living in lower socioeconomic or regional areas most affected (11). It is predicted that if current rates of childhood weight gain continue, prevalence of these conditions among Australian children will reach 33% by 2025 (12).

Research indicates that early intervention at or within a few months of birth can benefit obesity prevention in the first 1,000 days (2, 13–15). The World Health Organization's Commission on Ending Childhood Obesity (16) describes a continuum of care for the prevention, management and treatment of obesity among infants and children using a multi-strategy approach targeting the individual, family, community and public policy. Recent reviews support this approach, suggesting the use of interventions that include multicomponent (healthy eating, sleep, sedentary or screen-time, and physical activity or active play) guidance and support (17), and targeting system-level determinants of a child's diet and movement behaviors, such as caregiver behaviors, household and external environments, and food supply chains (18). The main influence of health behaviors in the early years is the family and home environment (19), therefore family-focused health services are well placed to provide this multicomponent support. In Victoria, Australia, this opportunity is available through the universal free Maternal and Child Health (MCH) service which provides 10 consultations between birth and age 3.5 years, with an uptake of 83–97% in the first 12 months (20).

While still an understudied area, expert consensus is emerging regarding the conceptualization of sustained implementation, especially clarifying the definition, developing an underpinning framework, and advancing measurement/assessment criteria (21–30). Sustained implementation is considered to have occurred when, “*after a defined period of time, the program/intervention/strategies continue to be delivered and/or individual behavior change (i.e., clinician, patient) is maintained, either as originally planned or with some degree of adaptation, while continuing to produce benefits for individuals/systems*” (27, 31). Sustained implementation, originally described as “institutionalization” (32) and more recently as “routinization” (33), “maintenance” (30) and “continuation beyond financial security” (28) is less frequently investigated in comparison to adoption and initial implementation, often due to budgetary and timeframe constraints (34).

Barker et al. (35) propose sustained implementation be a consideration during program development, small-scale replication, and real-world “at scale” implementation. This proposition appears justified given the number of programs/interventions/strategies implemented “at scale” that fail to be sustained long-term. In one of the earliest publications to examine the sustained implementation of public health programs, Scheirer (36) reported 40–60% were being implemented to some extent 1–6 years post program adoption. A multi-stage international literature search by Indig et al. (37) identified 40 public health interventions in high income countries (USA, Australia, Netherlands, Canada, UK, New Zealand, Finland) that showed reliable evidence of being implemented “at scale” between 1990 and 2014, of which 80% were still being implemented “to some extent” largely through institutionalization (55%) or commercialization (20%). A recent realist review (38) of nutrition and/or physical activity interventions implemented “at scale” (at a State or National level) within Australia since 2010, found four of the identified seven interventions (57%) were still being implemented 8 years post program adoption (one national and three in New South Wales).

As highlighted by Glasgow et al. (39) more than 20 years ago, numerous evaluated interventions are “lost in translation” because implementation is not sustained in real-world settings. Further, the “Determinants of Diet and Physical Activity” (DEDIPAC) Knowledge Hub study (40), informed by two umbrella reviews, reported a lack of research providing detail of implementation processes from the perspective of the health professional, practitioner or policy-maker, especially after completion of research projects. Understanding what factors impact sustained implementation is therefore essential to inform the, often significant, investments made by public health and government entities in developing and implementing programs “at scale” in real-world settings (41). While there is a sense of urgency to implement programs “at scale” in order to maximize their reach (42), it would appear that selection of programs is often based on availability and opportunity rather than proven efficacy or ability for sustained implementation (43).

The present study explored barriers and enablers influencing sustained implementation of the *INFANT Program* following the cessation of the state-wide prevention initiative, Healthy Together Victoria. Sustained implementation was defined as delivery of the *INFANT Program* (six three-monthly program sessions with first time parents of infants aged 3–18 months using a group-based format) between 2016 and 2017. Perspectives were obtained from trained *INFANT* facilitators, providing important insights into implementation processes, barriers and enablers experienced by health practitioners tasked with program implementation in “real-world” settings. Ethical approval for this study was obtained through Deakin University (HEAG-H 183_2014).

## Methods

### Program context

The *INFANT Program* is believed to be the first of its kind to address obesity risk behaviors in the first 1,000 days of life using a universally delivered service. Delivered in Australia, this is an efficacious, group-based program for first-time parents, comprising six 1.5-hour sessions delivered at three-monthly intervals when their infant is aged approximately 3, 6, 9, 12, 15 and 18 months (44) with positive health outcomes evident for mother and infant (45). The evolution of the *INFANT Program* from randomized controlled trial to small-scale community-level implementation (46) and the varying models of program implementation used (47) have been reported elsewhere. In 2014, the *INFANT Program* was included as a strategy within the state-wide prevention initiative, Healthy Together Victoria (HTV) (48). HTV operated across Victoria (2011–2016) to deliver a package of programs and strategies using a systems approach, with specific health promotion workforce funding and support provided to 14 local government areas (LGAs),^1^ based on socio-demographic indices and chronic disease risk factor prevalence. Due to national governance changes, funding ceased in 2015 (ahead of its scheduled 2018 end date) with the resultant cessation of HTV. Despite this early withdrawal of funding, a few LGAs continued to implement some of their activities, including the delivery of the *INFANT Program*. This provided an opportunity to investigate factors influencing the uptake and sustained implementation of the *INFANT Program* by LGAs, especially those previously receiving HTV funding.

### Study design

This study used a multi-site qualitative exploratory approach to facilitate an in-depth understanding of barriers and enablers to the sustained implementation of the *INFANT Program* within Victoria, Australia (49). This was considered a pragmatic and appropriate approach given the intent was to explore constructs to inform future examinations of the area. The researchers followed the Consolidated criteria for reporting qualitative studies (COREQ) checklist (50).

### Theoretical framework

The Consolidated Framework for Implementation Research (CFIR) (51) was selected as the underpinning theoretical framework as it offered the opportunity to explore a breadth of possible barriers and enablers across patterns of implementation (never, discontinued, ongoing). The CFIR comprises 37 constructs across 5 domains, each considered important for the adoption, implementation and embedding of interventions into routine practice (51) (Table 1). At the time of this study the CFIR was considered the most contemporary model available, underpinned by implementation research with practical application across diverse settings. Since this study was concluded, specific sustainability models have emerged, such as Integrated Sustainability Framework (ISF) (29). The use of the CFIR model to reflect elements of sustainability is however still considered relevant given the strong alignment between the constructs of the CFIR and ISF. The CFIR Guide Tool (CFIR Booklet (cfirguide.org) was used to develop survey and interview questions. (Table 1, Supplementary material 1). While the CFIR can be applied using a quantitative approach (52, 53), this study applied a qualitative approach as commonly used by others (54, 55).

**Table 1:** Consolidated framework for implementation research (CFIR) (51).

**Domain**	**Construct**	**Example questions used for surveys (S) /interviews (I)** **(see Supplementary material 1 for detail)**
Intervention characteristics	• Intervention source development and implementation decision-making process • Strength and quality of evidence to support choice of intervention • Relative advantage of implementing intervention versus an alternative • Adaptability of intervention to meet local needs • Trialability of intervention prior to implementation • Complexity and difficulty of implementation • Design quality and packaging of intervention • Costs associated with implementation	• What were the reasons why you attended the facilitator training? (S; I) • What did you know about the *INFANT Program* (if anything) before you attended the facilitator training? (I) • In your opinion, why do you think it was decided that the *INFANT Program* should/not be implemented in your area? (S; I)
Outer setting	• Patient needs and resources met in relation to implementation barriers/enablers • Cosmopolitanism (organization networks with other external organizations) • Peer pressure to implement intervention • External policy and incentives (mandates, strategies) to spread intervention uptake	• In your opinion, why do you think it was decided that the *INFANT Program* should/not be implemented in your area? (S; I) • What factors do you think helped /hindered the implementation of the *INFANT Program* in your area? (S; I) • How was the decision made that the program would/not be implemented in your organization? (I)
Inner setting	• Structural characteristics of the organization, such as maturity, age and size • Networks and communications (informal or formal) within organization • Culture, norms, values and basic assumptions of the organization • Implementation climate (receptivity, compatibility, relative priority, incentives) • Readiness for implementation (leadership engagement and commitment, available resources, access to knowledge, information incorporated into work tasks)	• Was the decision influenced by any other organizations implementing the *INFANT Program*, and if so, how? (I) • How does the *INFANT Program* fit within existing services within your organization? (I)
Implementer characteristics	• Knowledge and beliefs about the intervention and value placed on intervention • Self-efficacy/belief in own capabilities to implement intervention to achieve goals • Individual stage of change (level of preparedness to implement intervention) • Individual identification with the organization (commitment to organization) • Other personal attributes (learning styles, capacity, competency, motivation, etc.)	• What did you know about the *INFANT Program* (if anything) before you attended the facilitator training? (I) • How well did the training prepare you to implement the *INFANT Program* in your area? (I)
Implementation process	• Planning processes for implementation • Engagement strategies (with opinion leaders, champions, key stakeholders) • Executing according to implementation plan • Reflecting and evaluating (qualitative and quantitative feedback on progress)	• How was the *INFANT Program* planned and implemented in your area? (I) • How have you gone about evaluating the *INFANT Program* in your organization? (I)

### Data instrumentation

Open-ended questions within the surveys were used to explore barriers and enablers to the sustained implementation of the *INFANT Program* following facilitator training, with follow-up interviews to explore findings in more depth (Supplementary material 1). Participants completed a 15-minute online survey regarding their perspectives of the *INFANT Program* training, reasons for attending training, intentions of program delivery after training, and tailored questions depending on the pattern of program implementation (never, discontinued, ongoing). The survey was structured according to pattern of implementation, with tailored questions framed by the CFIR domains (51) to identify enablers and barriers to ongoing (sustained) implementation. Questions comprised open-ended and 7-point Likert scale (completely disagree-completely agree) responses. Follow-up 30–45 min audio-recorded telephone interviews were conducted with consenting survey participants to explore survey responses further. Interview questions asked participants to reflect on organizational decision-making about the planning process, resourcing and support for the implementation of the *INFANT Program* after completing the face-to-face training.

### Data collection

All Victorian-based staff who had completed the *INFANT Program* facilitator training between 2013 and 2017 (*n* = 88) were contacted, using email contact details provided during training registration. Those contacted were invited to complete an online survey and an optional telephone interview. Those consenting to an interview were contacted directly by PL to schedule a convenient date and time for the interview. All interviews were conducted by PL using a semi-structured interview guide, ranging in duration from 21–47 min. Audio-recorded interviews were transcribed verbatim by an external agency. No incentives were offered to participate in the study.

Of the 88 Victorian-based *INFANT Program* trainees, two were not contactable, four were on leave and 16 had moved to other positions, resulting in a final sample size of 63 participants, representing 16 LGAs (six regional and 10 metro) at various stages of implementation (never implemented *n* = 6; discontinued implementation *n* = 5; ongoing implementation *n* = 5). Thirty-one participants completed the online survey, with 11 consenting to follow-up interviews, representing 14 LGAs across all patterns of implementation (never implemented *n* = 4; discontinued implementation *n* = 5; ongoing implementation *n* = 5).

### Data analysis

Qualitative data analysis was underpinned by a contextualist epistemology, where knowledge emerges from and is situated within the context of the data (56). As the interpretation of qualitative data can be influenced by the roles and backgrounds of the researchers, these are made explicit. All researchers have a health qualification and work within a research context. At the time of the study MW was a research assistant with nutrition experience, and PL, RL, and ST were postdoctoral researchers with experience in the implementation of public health nutrition interventions at a community level. MW, ST, PL, and RL had no involvement in the development of the *INFANT Program*. RL had specific involvement in evaluating the small-scale community implementation of the *INFANT Program*. KDH and KJC are chief investigators of the *INFANT Program*, responsible for its development, randomized control trial, small-scale community implementation, and ongoing evaluation.

A reflexive thematic analysis approach, as described by Clarke et al. (57), was undertaken using open-ended survey responses and interview transcripts to determine shared meaning underpinned by the CFIR domains (51). Data were coded deductively (informed by the CFIR framework) and inductively (to identify other codes) using NVIVO v12 (QSR International, Melbourne, Australia (58). A sub-sample of interviews was coded independently by three co-authors (PL, ST, and MW), followed by discussion regarding interpretation and application of the coding framework. All coding was completed by MW. NVIVO coding summaries were used for case comparison analysis to identify similarities and differences between barriers and enablers for different patterns of implementation across the LGAs, namely, never, discontinued, and ongoing (sustained) implementation. Consensus on final theming was developed in agreement between PL, RL, KDH, and KJC. As an exploratory study with a small sample size, data saturation was not a consideration.

## Results

### Description of participants

Thirty-one participants completed the online survey, with 11 consenting to follow-up interviews. All participants were female, mainly between the ages of 40–59 years (71%). Most participants were Maternal and Child Health Nurses (48%), followed by dietitians (2.5%), and in part-time roles (68%). Across all LGAs, the main reason for attending *INFANT Program* training was to become skilled to implement the program. The majority of participants attended training to take on the role of program facilitator (88%), and “mostly” and “completely” agreed that training provided the necessary knowledge (81%) and confidence (74%) to implement the *INFANT Program* (Table 2).

**Table 2:** Descriptive data of study participants by pattern of implementation.

**Study participant descriptor**		**Pattern of implementation (*****n*** = **31 survey responses)**			
		**Total**	**Ongoing**	**Discontinued**	**Never**
Gender	Female	31	15	11	5
Age	20–29 years	3	3	0	0
	30–39 years	4	3	1	0
	40–49 years	12	5	6	1
	50–59 years	10	4	3	3
	60+ years	2	0	1	1
Profession	Maternal child health nurse	15	8	5	2
	Dietitian	8	6	0	2
	Health promotion officer	2	1	1	0
	Early childhood professional	**2**	0	1	1
	Social worker	1	0	1	0
	Early intervention worker	1	0	1	0
	Children and family resource officer	1	0	1	0
	Bicultural families and children officer	1	0	1	0
Full/Part-time	Full time	10	5	3	2
	Part-time	21	10	8	3
Years in role	< 5 years	7	5	2	0
	5–10 years	9	6	3	0
	11–15 years	4	2	1	1
	>15 years	11	2	5	4
Reason for attending training (multiple options)	Intention to deliver	16	7	8	1
	Gain additional knowledge	11	6	3	2
	Learn about the program	19	10	4	5
	Personal professional development	10	5	3	2
	Organization already delivering	10	8	2	0
Program LGAs	HTV-funded (7 LGAs)	21	11 (2 LGAs)	9 (4 LGAs)	1 (1 LGA)
	Non-HTV funded (7 LGAs)	10	4 (3 LGAs)	2 (1 LGA)	4 (3 LGAs)

### Patterns of implementation

Online survey participants represented 14 LGAs across all patterns of implementation (never implemented *n* = 4; discontinued implementation *n* = 5; ongoing implementation *n* = 5). Of these LGAs, 11 participants consented to interviews representative of all patterns of implementation (never implemented *n* = 3; discontinued implementation *n* = 4; ongoing implementation *n* = 4). All patterns of implementation were evident across regional and metro LGA locations. Regional LGAs (*n* = 5) reported *n* = 1 as never implemented; *n* = 2 with discontinued implementation; and *n* = 3 with ongoing implementation. Metro LGAs (*n* = 9) reported *n* = 3 as never implemented; *n* = 3 with discontinued implementation; and *n* = 2 with ongoing implementation. Of the 14 LGAs, seven (3 regional; 4 metro) had received specific health promotion workforce funding through the HTV initiative (never implemented *n* = 1; discontinued implementation *n* = 4; ongoing implementation *n* = 2), and seven were non-HTV funded (never implemented *n* = 3; discontinued implementation *n* = 1; ongoing implementation *n* = 3) (Table 2).

### Barriers and enablers to sustained implementation

Mapping identified barriers and enablers to the CFIR (Supplementary material 2) revealed the inner and outer settings and implementation process to be of greatest influence.

#### Inner setting

Organizational implementation climate and readiness for implementation were most frequently described by participants. LGAs that had never implemented the *INFANT Program* felt that the “*timing was not right*”, with a lack of agreement between organizations regarding the implementation approach. These LGAs also reflected on limited leadership engagement and the lack of a program “champion”. A lack of management support was the main barrier cited by the HTV-funded LGA that had never implemented the *INFANT Program* whilst a lack of funding and availability of staff to coordinate and deliver the program were main barriers cited by non-HTV funded LGAs with no implementation—“*I'm sure it can be done it was just too hard for us without resources at our disposal” [Never implemented, metro LGA]*.

LGAs with discontinued or ongoing implementation felt the *INFANT Program* was highly compatible with existing services and a priority. LGAs with discontinued implementation reflected that the program was competing with other priorities, and in some cases, other programs. The main barrier cited by all four HTV-funded LGAs that had discontinued implementation was the cessation of funding—“*(HTV) funding ceased, and management deemed it [the INFANT Program] was no longer needed” [Discontinued implementation, metro LGA]*. The non-HTV funded LGA that had discontinued implementation cited a lack of management support and poor program attendance as the main barriers.

Only LGAs with ongoing implementation described consideration of sustained implementation at the start—“*We made the decision at the start that it [INFANT Program implementation] was going to keep going beyond the funding time… we needed to embed it into services that we have already” [Ongoing implementation, regional LGA]*. LGAs with ongoing implementation also mentioned the importance of establishing organizational connections prior to undertaking the training to achieve early buy-in. For both HTV-funded and non-HTV funded LGAs, management support was cited as the main enabler to implementation.

Available implementation capacity and resources was described as a limiting factor by LGAs across all patterns of implementation, especially when attendance rates were low, and even if program delivery was incorporated into staff roles as “*once (HTV) funding stopped, the positions stopped” [Discontinued implementation, regional LGA]*. LGAs with ongoing implementation described how staff capacity had been created through the allocation of health promotion hours within existing staff roles, clarifying role responsibilities between partner organizations (such as referrals by maternal child health, and scheduling by community health), and establishing designated administration support to streamline enrolment, reminder notifications, and securing venues. LGAs with discontinued or ongoing implementation described strong organizational engagement, especially between dietetic and maternal child health services.

#### Outer setting

Across all patterns of implementation, LGAs described the *INFANT Program* as meeting a community need, complementing and strengthening the universal Maternal and Child Health (MCH) Service offered across Victoria. LGAs that had never implemented suggested that the program be promoted more as “*people haven't any idea of what it is or the benefits” [Never implemented, metro LGA]* and commented on the need to consider more contemporary approaches to program delivery in line with current technology -“*introducing the electronic form of it… because most people have smartphones” [Never implemented, metro LGA]*.

All LGAs expressed a desire to be connected with local organizations to assist with program recruitment, implementation and to provide “*positive feedback from another organization already running the program” [Ongoing implementation, regional LGA]*. Access to the *INFANT Program* research team for implementation guidance was a valued support by LGAs with discontinued or ongoing implementation.

LGAs with discontinued or ongoing implementation suggested better alignment between funding and policy directives, with recurrent funding, resourcing and monitoring to enable sustained implementation—“*It would be lovely to just be able to do it in a fully funded, dedicated way… through state or federal funding… in the same way that other services are provided then you can dedicate staff to it” [Discontinued implementation, regional LGA]*.

Across all LGAs, two main models of program implementation were apparent, one led by the MCH team (based within local government) and the other a partnership between the MCH team and dietitians (based within community health). All LGAs with ongoing implementation had a partnership model in place.

#### Implementation process

While LGAs across all patterns of implementation described the *INFANT Program* as aligning to existing services and having the potential to replace ad hoc group information sessions, only LGAs with ongoing implementation spoke about integration of the program into service provision. Examples included delivery of the first *INFANT Program* session as part of existing New Parent Groups, enrolling participants into all sessions with automated reminder notifications and opt-out consent (rather than individual session enrolment) and offering “open” groups so participants could attend any missed sessions. LGAs with discontinued and ongoing implementation both made adaptations to program delivery, predominantly delivering four of the six sessions (3, 6, 9, and 12 months) given the high attrition rates at the 15 and 18 month sessions. LGAs all described undertaking some form of program evaluation, expressing concerns about unrealistic targets, what data to collect, and participant burden.

The importance of engagement and involvement of key partner organizations and stakeholders was evident across all patterns of implementation. LGAs that had never implemented the *INFANT Program* echoed their feedback regarding a lack of consensus by partner organizations about the appropriate implementation approach, with no opinion leaders or program champions. LGAs with discontinued implementation spoke of the need for a designated implementation team so that implementation was not in addition to existing workloads—“*… to do it properly… get all the admin done… all that really needs a designated team. We were a bit caught between what we were already doing…” [Discontinued implementation, regional LGA]*. LGAs with ongoing implementation described the partnership between dietetic (community health) and maternal child health (local council) as ideal for the implementation of the *INFANT Program*, but that this required a shared understanding and clarity regarding implementation roles and responsibilities. Engagement with “external change agents” was suggested across all patterns of implementation and included the promotion and/or extension of the *INFANT Program* into childcare centers, playgroups, and ante-natal groups.

#### Intervention characteristics

Across all patterns of implementation, LGAs were aware of the *INFANT Program* prior to attending training, through professional conferences, colleagues and management, and as an endorsed HTV program. All LGAs considered the program to be evidence-based with strong research outcomes, offering a relative advantage to the organization in terms of alignment with current MCH services, and providing consistency of information to parents. LGAs with ongoing implementation regarded the program as “value-adding” by providing a more structured approach, replacing ad hoc group information sessions “*to allow time to deliver INFANT Program which covers these topics plus more” [Ongoing implementation, metro LGA]*. LGAs with discontinued or ongoing implementation described similar complexities in relation to scheduling of sessions with similar aged infants, and venue availability and costs. Regional LGAs in particular were challenged by small birth rates and large geographical distances which limited attendance rates and group size. Costs associated with the *INFANT Program* were described in terms of implementation capacity, and not in relation to accessing training or program resources.

All LGAs described the need to consider flexibility with implementation of the *INFANT Program* to meet community needs, such as providing more visual images or using an interpreter for different cultural groups, tailoring delivery for groups with mixed age groups, and most commonly, providing fewer sessions given the low attendance rates at the 15 and 18 month sessions. One non-HTV funded LGA that had never implemented the program was concerned about how much flexibility and adaptation could be applied before impacting on program fidelity—“*[I'm} concerned that adapting the program by combining sessions or offering them in other formats does not have the evidence base” [Never implemented, metro LGA]*.

LGAs across all patterns of implementation considered the training and website resources to be of high quality. LGAs with ongoing implementation described the training as enhancing group facilitation skills. LGAs with discontinued implementation felt the training had reinforced existing knowledge, enhancing levels of confidence to deliver program sessions. LGAs that had never implemented expressed a need for specific implementation guidance and examples of how LGAs had implemented the program, especially where this had occurred without additional funding.

#### Implementer characteristics

Across all patterns of implementation, study participants considered themselves to possess the appropriate knowledge and beliefs to implement the *INFANT Program*, describing the delivery of infant feeding and active play information to parents as “*our bread and butter”* and “*part of our core work” [Ongoing implementation, metro LGA]*. LGAs with both discontinued and ongoing implementation described the training as enhancing levels of confidence to present the content and facilitate the group discussion in a different way, with “*a greater focus on active listening” [Ongoing implementation, regional LGA]*.

## Discussion

This study explored barriers and enablers to sustained implementation of an early childhood health behavior program for parents, the *INFANT Program*, during small scale implementation in Victoria, Australia, from the perspective of trained *INFANT* facilitators. Challenges regarding complexities of program implementation were apparent across all patterns of implementation, with requests for specific implementation guidance and connections with other LGAs achieving successful implementation. The main differences between LGAs with ongoing and discontinued implementation related to the “inner and outer setting” and “implementation process”, specifically, funding availability, organizational resourcing and capacity, organizational management support and endorsement, integrating implementation into routine practice, establishing early buy-in and role clarity with partner organizations, and planning for sustained implementation from the start.

The enablers and barriers identified in this study are similar to those reported in the literature and can therefore be considered to have relevance to other health promotion interventions. Muellmann et al. (40) describe five main enablers, relevant to multi-level interventions and policies promoting healthy eating and physical activity, namely, stakeholder networks, structures in settings, continued funding and political support, standardized training of staff with detailed implementation protocols, and socio-cultural tailoring of content to fit the needs and context of the targeted population. In addition to these enablers, Mikkeslen et al. (59) report the need for capacity building of health professionals across health, education and community settings, including pre-service and in-service training, so that implementation activities continue after any research support concludes. Similarly, systematic reviews of health promotion (28), community-based obesity prevention (23), healthcare (22), schools and childcare services (60) and public health (61) interventions highlight the importance of several recurring enablers, namely: strategic planning, program alignment, integration into existing programs and policies, accessing new/existing money to facilitate sustainment, leadership prioritization and support to mobilize implementation, adequate human resourcing, workforce development and capacity building regarding implementation planning and evaluation, systematic adaptation to enhance compatibility of the intervention with the organization, monitoring progress and demonstrating effectiveness, and establishing organizational partnerships.

The provision of external funding through the HTV initiative was a key catalyst for *INFANT Program* implementation, however four of the seven HTV-funded LGAs discontinued implementation once HTV funding ceased. LGAs with ongoing implementation of the *INFANT Program* utilized strategies that were not reliant on external funding support, in particular, creating staff capacity through the allocation of health promotion hours within existing staff roles, and establishing a partnership model for implementation between community health dietetic and maternal child health services. Cross-disciplinary and cross-organizational partnerships, with a shared agenda, can frequently add tangible resources to the implementation process (62). Investment in organizational capacity and infrastructure creates a foundation for the intervention activities to continue if/when external resources, such as a research team or government agency, are discontinued (59).

For both HTV-funded and non-HTV funded LGAs, management support was cited as the main enabler to ongoing implementation. The role of leaders and transformational leadership in supporting sustained implementation is well documented (23, 52, 63, 64) in the form of policy and reward systems, organizational decision-makers, and community champions. Effective leaders can mobilize capacity and collaboration, frequently overcoming organizational indifference or opposition to a new intervention. Leaders are also instrumental in generating program awareness and securing ongoing investment. The uptake of the *INFANT Program* by HTV-funded LGAs is indicative of the leadership endorsement of the program as part of the HTV initiative. With the cessation of the HTV initiative, the loss of this endorsement and the removal of funding resulted in many HTV-funded LGAs discontinuing their implementation of the *INFANT Program*.

Early consideration of sustained implementation was identified as a common strategy by LGAs with ongoing implementation of the *INFANT Program*. This view complements the growing consensus that sustained implementation should be considered from the beginning of the implementation process, with dedicated planning to define program components and determinants to inform appropriate implementation strategies (28). An integral part of this early planning phase includes dedicated exploration of an organization's readiness in terms of commitment and capability for implementation, which has now been incorporated as a consideration during *INFANT* facilitator training. When organizational readiness for change is high, organizations display greater initiation, persistence and cooperation to achieve successful implementation (53, 65). In a recently updated systematic review, Miake-Lye et al. (66) mapped organizational readiness assessment instruments to the CFIR, and identified “readiness to implementation” as the most commonly reported construct.

The challenge of fidelity and adaptation was identified as a barrier by LGAs that had never implemented the *INFANT Program* post facilitator training. These LGAs described being unsure what degree of adaptation would be possible without impacting program fidelity and were seeking specific implementation guidance. The assumption that intervention effects lessen if implemented “at scale” without careful adherence to research protocols has been challenged by Chambers et al. (34) who suggest that this constrains the intervention “fit” (compatibility) within the given context and positions sustained implementation as “the endgame”. They propose that sustained implementation be a consideration throughout the implementation process to accommodate adaptation so that the intervention becomes integrated into the local context (34). To facilitate a more precise understanding of adaptations made to the *INFANT Program* when implemented in real-world settings, comprehensive documentation using the Framework for Reporting Adaptations and Modifications-Enhanced (FRAME) (67) has subsequently been incorporated into the *INFANT* Effectiveness-Implementation Trial (68) to inform the timing, context and process for adaptation to facilitate sustained implementation. Capturing intervention adaptation is a key inclusion to establish the degree to which intervention components are modified for organizational compatibility without jeopardizing intervention outcomes.

De-implementation strategies are likely to become an important consideration for the sustained implementation of the *INFANT Program*, as LGAs all commented about competing organizational priorities and the need for flexibility to meet community needs, such as tailoring session content and/or delivery mode. Acknowledging that intervention adaptation, whether organic or planned, occurs and is beneficial to sustained implementation, elicits an additional consideration of de-implementation of intervention strategies /components considered no longer compatible or effective (30). De-implementing detrimental or redundant practices is distinct from implementing evidence-based practices, and is considered more difficult, requiring more intense strategies. Norton and Chambers (69) propose four types of de-implementation actions—removing, replacing, reducing or restricting the use of a specific intervention strategy /component. They suggest that future research identify and map specific sustainment barriers to appropriate de-implementation strategies, as is done for implementation strategy development, as well as optimal timeframes and pace at which de-implementation should occur, to mitigate potential harm or unintended negative consequences.

### Implications for the *INFANT Program*

This study has provided the opportunity to investigate sustained implementation of the *INFANT Program* during small scale community-level implementation. The factors influencing sustained implementation of the *INFANT Program* highlight a number of organizational (inner) and system-level (outer) barriers and enablers that are interconnected around prioritization and endorsement, leadership and management support, human and financial resourcing, and capacity building.

These study findings have contributed important insights in preparation for large scale implementation across Victoria and its associated effectiveness-implementation trial (68). Findings have informed the refinement of intervention characteristics, namely, online facilitator training and refresher training and a community of practice (collaborative online forum), and delivery as four group sessions (3–12 months) supplemented with app-based messages (birth to 18 month). Post COVID-19 and the emergence of telehealth, virtual (online) group delivery has also become a consideration for future exploration. Findings have also informed the selection of specific implementation strategies to support adoption and sustained implementation of the *INFANT Program* across local government areas. Using the Expert Recommendations for Implementing Change (ERIC) compilation (70), key strategies have been selected to address barriers to sustainability, namely:

- Accessible, incentivised online training, an online community-of-practice, and a Statewide training coordinator role to build capacity of organizational implementers
- Statewide implementation coordinator role to facilitate implementation planning with local organizations, and capture intervention modifications
- Early assessment of organizational implementation readiness and timely provision of appropriate implementation support, such as implementation case studies available on the *INFANT Program* website [Deliver *INFANT* | *INFANT* (infantprogram.org)]
- Leveraging key state and local level policy opportunities to embed *INFANT Program* delivery, such as the Victorian government's *Healthy Kids, Healthy Futures* 5-year action plan (71), and the *Victorian Public Health and Wellbeing Plan 2019–2023* (72).

Furthermore, the *INFANT* effectiveness-implementation trial (68) will include an evaluation timepoint at 24-months post facilitator training which will assess program sustainability using the Program Sustainability Assessment Tool (self-administered surveys) (73) with follow-up in-depth interviews.

### Implications for research

Since completion of this study, Shelton et al. have developed the Integrated Sustainability Framework (ISF) (29) which proposes key multilevel factors needed for sustained implementation of interventions across settings and contexts, namely, outer contextual characteristics (policy environment and funding, organizational partnerships), inner contextual characteristics (organizational infrastructure and support, leadership and program champions, funding), implementation processes (e.g., recruitment, training, strategic planning and communication, evaluation), characteristics of interventionists (role commitment and motivation, self-efficacy, payment), and intervention characteristics (perceived benefit/need for program, program fit and adaptability). The ISF factors are similar to those identified in the CFIR used in this study (51). Future research utilizing the ISF would be useful to advance the application of a specific framework to guide implementation sustainability research.

### Strengths and limitations

This study used a recognized theoretical framework within the field of implementation science, the Consolidated Framework for Implementation Research (CFIR) (51). Described as a determinant framework, mapping identified barriers and enablers to the CFIR advances understanding of how and why sustained implementation occurs across multiple levels of influence using a systems approach (74). While a relatively small sample size, the response rate was high (49.2%) for this type of research with almost equal representation of LGAs across all patterns of implementation (never, discontinued and ongoing), reducing the risk of social bias. The online survey questions were informed by the literature. Closed response options may have limited participant responses, however, open-ended response fields were also provided to elaborate on survey responses, and participants were offered an optional interview opportunity to expand on responses. This study collected data in 2017 from *INFANT Program* facilitators who completed their training between 2013 and 2017 with training completion dates evenly distributed across patterns of implementation, therefore any effects of potential participant recall bias would have been similarly distributed.

## Conclusion

This study provides important insights into the barriers and enablers to the sustained implementation of an evidence-based intervention (the *INFANT Program*) during small scale community-level implementation. The opportunity to gain insights on real-world implementation prior to delivery at-scale is rare, with decisions to scale-up interventions frequently occurring without adequate evidence of effectiveness and/or sustainment (43). The authors therefore contend that the pre-requisite for scale-up of a population health intervention is not just proof of effectiveness (75) but also proof of sustained implementation at the local/organizational level. In addition, assessment of implementation readiness should occur beyond the stages of adoption and early implementation to inform strategies that support sustained implementation. The use of hybrid type 2 effectiveness-implementation trials is therefore strongly recommended to achieve such concurrent evaluation (68, 76).

The factors influencing sustained implementation of the *INFANT Program*, predominantly organizational and system-level barriers and enablers, have broad transferability given their remarkable similarity to those identified for health promotion interventions implemented across the world, in healthcare, education and community settings. This study is a reminder that sustained implementation requires investment, effective governance, partnerships and supportive systems. These should be fundamental inclusions when planning “at scale” intervention delivery to optimize opportunities to integrate intervention components into routine practices and policies thereby sustaining implementation.

## Data availability statement

The raw data supporting the conclusions of this article will be made available by the authors, without undue reservation.

## Ethics statement

The studies involving human participants were reviewed and approved by Deakin University Human Ethics Advisory Group-Health (HEAG-H 183_2014). The patients/participants provided their written informed consent to participate in this study.

## Author contributions

PL, RL, and KJC conceived and designed the study. PL obtained study ethics and undertook recruitment and study interviewees. ST and MW analyzed the data with cross-checking by PL. PL led the manuscript writing with input from all authors. All authors approved the manuscript for submission.

## Funding

The *INFANT* RCT trial was funded by a NHMRC grant (APP425801). Additional funds were supplied by the Heart Foundation Victoria and Deakin University. The *INFANT* Follow-up Study of outcomes at 3.5 and 5 years was funded by a NHMRC grant (APP1008879). RL held an NHMRC ECR Fellowship (#1089415) when conducting the small-scale implementation study. During this current study, PL and ST were funded through the NHMRC CRE for Early Prevention of Obesity in Childhood (EPOCH). KDH is supported by a Heart Foundation Future Leader Fellowship (#105929). The *INFANT* Effectiveness-Implementation Trial is funded by a NHMRC Partnership Grant (APP1161223) and VicHealth.

## Conflict of interest

The authors declare that the research was conducted in the absence of any commercial or financial relationships that could be construed as a potential conflict of interest.

## Publisher's note

All claims expressed in this article are solely those of the authors and do not necessarily represent those of their affiliated organizations, or those of the publisher, the editors and the reviewers. Any product that may be evaluated in this article, or claim that may be made by its manufacturer, is not guaranteed or endorsed by the publisher.

## Footnotes

^1^Victoria, Australia, comprises 79 local government areas (LGAs) [municipalities] with elected councils providing governance in relation to local laws across a range of community services.
