# Supplementary material for: Factors contributing to the sustained implementation of an early childhood obesity prevention intervention: The INFANT Program
Source: Front Health Serv. 2022 Nov 25;2:1031628. doi: 10.3389/frhs.2022.1031628 (PMC10012774; doi:10.3389/frhs.2022.1031628)
Supplement: Supplementary file 1 [file Data_Sheet_1.PDF]

Supplementary File1: Mapping of INFANT survey and interview questions to Consolidated Framework for Implementation Research (CFIR) domains

Survey questions:	Interview questions:	Consolidated Framework for Implementation Research (CFIR) – Damschroder et al 2009
**Why did you attend the INFANT program training?**<br>Options: intending to implement; already implementing and training more facilitators; gain additional knowledge about infant feeding; learn about the INFANT program; professional development; other<br><br>**Is the INFANT Program currently running in your area?**<br>Yes, No - but has been previously, No - never run	**What did you know about the INFANT program (if anything) before you attended the facilitator training?**<br>Prompts: evidence-based, trialled, being run successfully elsewhere…<br><br>**What were the reasons why you attended the facilitator training?**<br>Prompts: personal PD, identified organisation need….<br><br>**How well did the training prepare you to implement IFANT in your area?**<br>Prompts: what was most useful, anything not covered; additional support that would have been useful?<br><br>**What is/was your role in implementing the INFANT program in your area?**<br>Options: program administrator, program coordinator, program facilitator, other	Intervention characteristics<br>- evidence strength and quality; intervention source relative quality; adaptability; trialability; design quality and packaging; cost; complexity
Yes:<br>**In your opinion, why do you think it was decided that the INFANT Program should be implemented in your area?** [open ended response]<br>**What is your current role in implementing the INFANT program?**<br>Options: program administrator, program coordinator, program facilitator, other<br><br>No-but has been previously:<br>**In your opinion, why do you think it was decided that the INFANT Program should no longer be implemented in your area?** [open ended response]<br>**What was your role in implementing the INFANT program?**<br>Options: program administrator, program coordinator, program facilitator, other<br><br>No-never run:<br>**In your opinion, why do you think it was decided that the INFANT Program should not be implemented in your area?** [open ended response]	**After attending the training, what were the reasons that the program was/was not implemented in your area?**<br>Prompts: meeting community need; available funding and resources, incentives eg: performance measures, alignment with internal/external policies, regulations or guidelines, management support, program champion; other org priorities; communication…<br><br>**How was the decision made that the program would/not be implemented in your organization?**<br>Prompts: were staff involved in the decision; how did the staff feel about the decision<br><br>**Was the decision influenced by any other organizations implementing the program, and if so, in what ways?**<br><br>**NEVER IMPLEMENTED INTERVIEWS ONLY**<br>What do you think would be needed for the INFANT program to be implemented in your area?<br>**END OF INTERVIEW QUESTIONS FOR 'NEVER IMPLEMENTED PROGRAM'**	Inner setting<br>- tension (need) for change; organisational characteristics/compatibility/culture/incentives and rewards; networks and communications; goals and feedback (reporting requirements); relative priority; implementation climate; implementation readiness (leadership engagement; resources; knowledge)<br><br>Outer setting<br>- meeting client needs; external policies and incentives; peer pressure; cosmopolitanism<br><br>Map to relevant CFIR as mentioned
Yes / No-but has been previously:<br>**In your opinion, which of the following factors enabled the INFANT Program being implemented in your area?** (Select as many as apply)<br><br>No-but has been previously / No-never run<br>**In your opinion, which of the following factors contributed to the INFANT Program no longer / not being implemented in your area?** (Select as many as apply)<br><br>➢ Availability of funding to implement the Program<br>➢ Evidence to support the decision to implement the Program<br>➢ Availability of staff to deliver Program sessions<br>➢ Confidence of staff to deliver Program sessions<br>➢ Availability of staff to co-ordinate the Program	**How was the program planned and implemented in your area?**<br>Prompts:<br>What was your role in implementing the INFANT program?<br>Who was involved in the planning process?<br>What was considered in the planning process? Eg: resources<br>How do you recruit and retain participants in the program? Do you advertise the program, where and how?<br>Who delivers the sessions? Level of confidence?<br>What format? Age cohort of groups?<br>Was the program changed in any way to address the needs /preferences/barriers experienced by participants; and in what ways?<br><br>**How does the program fit within existing services within your organisation?**<br>Prompts:	Implementation process – planning, engaging stakeholders, executing, evaluating<br><br>Characteristics of facilitators – knowledge/beliefs, self-efficacy (confidence), personal attributes, ID with organisation, implementation readiness (stage of change)<br><br>Intervention characteristics – design quality and packaging; complexity; adaptability; relative advantage

➢ Management support for the implementation of the Program<br>➢ Alignment of the Program to the priorities of the organisation<br>➢ Ability to incorporate the Program as routine practice within the organisation<br>➢ Ability to recruit parents to the Program<br>➢ Ability to maintain attendance of parents to the Program sessions<br><br>Yes / No-but has been previously<br>**Were there any other factors that helped with the implementation of the INFANT Program in your area?** [open ended response]<br><br>No-but has been previously / No-never run<br>**Were there any other factors that contributed to the INFANT Program no longer / never being implemented in your area?** [open ended response]	What kinds of changes were needed so that the program could be implemented within your organisation e.g.: changes in scope of practice, to organisational policies or information systems?<br>Who decided whether changes were needed and what was the process for making these changes? Was there support to make these changes? In retrospect, were there changes you wish had/had not been made?<br>Is the program integrated as part of routine practice or is it still seen as a departure from current practices; and in what way?<br>What would you consider to be the key strengths/advantages of implementing the program in your area? Key disadvantages?<br><br>**How have you gone about evaluating the program in your organisation?**<br>Prompts:<br>Does your organisation have performance targets/goals related to the implementation of the program? What are they? How are they monitored? Are any feedback reports compiled, and if so, to whom are they disseminated?<br>Do you think the program has been effective in your community; in what ways; how do you know? (equity and numbers reached; retention rates; parental receptivity; impact – attitude, knowledge behaviour changes; broader community impacts eg: partnerships)	Inner setting – goals and feedback<br>Outer setting – patient needs and resources<br>Implementation process – reflecting and evaluating
Yes / No-but has been previously<br>**To the best of your knowledge, which of the following options best applies to your area?**<br>• Implementation of the INFANT Program is likely to continue<br>• Implementation of the INFANT Program is NOT likely to continue<br>**In your opinion, why do you think your organisation has made this decision?** [open ended response]<br><br>No-but has been previously / No-never run<br>**To the best of your knowledge, which of the following options best applies to your area?**<br>• Implementation of the INFANT Program is likely to occur in the future<br>• Implementation of the INFANT Program is NOT likely to occur in the future<br>**In your opinion, why do you think your organisation has made this decision?** [open ended response]	**What are the future plans for the implementation of the INFANT Program in your community?**<br>Prompts<br>Will the program continue to be implemented? Why/not?<br>Are there changes to the program that are needed so it can continue to be implemented (make it more sustainable)?<br><br>**In summary, what key lessons would you share regarding implementing the program?**<br>For organisations planning to/currently implementing programs?<br>For researchers planning/developing future programs?<br>For policy makers supporting future programs?	Map to relevant CFIR as mentioned
