# Supplementary material for: Factors contributing to the sustained implementation of an early childhood obesity prevention intervention: The INFANT Program
Source: Front Health Serv. 2022 Nov 25;2:1031628. doi: 10.3389/frhs.2022.1031628 (PMC10012774; doi:10.3389/frhs.2022.1031628)
Supplement: Supplementary file 2 [file Data_Sheet_2.PDF]

Supplementary File2: Coding of survey and interview responses by pattern of implementation to Consolidated Framework for Implementation Research (CFIR) domains

Consolidated framework for Implementation Research (CFIR) – Damschroder et al 2009	Pattern of Implementation		
	Never implemented	***Discontinued implementation***	***Ongoing implementation***
**INTERVENTION CHARACTERISTICS**			
Intervention source	One of the initial researchers…did a presentation at the maternal and child health conference and was talking about upcoming facilitator training [metro LGA]<br>I worked at one of the pilot program health centres, so I was aware that it was going on and I was aware that it was a good thing [metro LGA]	I sat in on one of the sessions and said, "This goes for longer than the initial introduction of solids, and we'd like to fund this" [metro LGA]<br>Through the Healthy Together Victoria initiative, we looked at a few different programs and ran the INFANT program [metro LGA]	I heard about the INFANT Program through one of the dieticians who work at [another LGA] because they were doing it as part of their Healthy Together Victoria program [regional LGA]<br>I knew a little bit. I used to recommend the website before I was a facilitator [metro LGA]
Evidence quality and strength	We follow the Australian Guide to Healthy Eating; they're our guidelines for educating families. The INFANT program aligns with that [metro LGA]<br>There were good things coming out of the evaluation. I just had in my mind that it would be a good thing to establish a program that's been built from the evidence [metro LGA]	Our manager was aware of the research around the program and the context that it was coming from and the benefits of intervening early. So yes we were aware of the benefits of it and the research context [regional LGA]	The program is evidence based.... we know that if that advice is delivered, that it actually changes behaviour... we know that from all the research [regional LGA]
Relative advantage	[During consultations] I'm able to refer them [parents] to the [INFANT program] website, I think that's been really useful [metro LGA]<br>From the start I said this is a program that would align well with working with maternal and child health and sessions for new parent's groups [metro LGA]	It was really, really well received.... a lot of social support went on I thought. It was nice to be able to offer them a combination of support. It was a good way to connect mums [regional LGA]	I think it was a value adding exercise .. a more structured approach, more targeted messages for the {new mothers} group [regional LGA]<br>I think that having a framework to work from, rather than just doing some sort of one-off session is what was really attractive for us, and it was a real gap for us [regional LGA]<br>The INFANT Program has replaced our Introduction to Solids group and our Fussy Feeders group. So we've been able to get rid of those two groups to then allow the time in the centres to deliver INFANT Program which covers those topics plus more [metro LGA]
Adaptability / Trialabilty	At the training, it talked about it being the 6 session program, and I remember asking, at the time, whether there was any flexibility around that, because I didn't think that I'd have a hope of being able to coerce the council into setting it up as six sessions [metro LGA]<br>What would help us is to know how better to deliver it... trying to provide access to the needier people in the community ...... [metro LGA]	We found it very Anglo-Saxon. The booklets and everything... Our community actually needs hands on... a lot more visual images rather than discussion. We would have to be very conscious of what images and what we showed them... 80 percent of our population are people from overseas [metro LGA]<br>I've still got a lot of stuff from INFANT on my desktop that I use since the program has stopped …. that I will show them [parents]. If nothing else, I have personally been able to get a lot out of it and use it for my [MCHN] practice [metro LGA]	It has taken a little bit of planning just because a lot of our key groups they've got a range of ages. If I do a 3month session I could be looking at a child who's two months to about eight months. So there's quite a range in there. So yes, I do have to tailor it sometimes depending on the age of the kids that are in the group [regional LGA]<br>We just found that our engagement with the 15 and 18month sessions really dropped off after that 12month sort of mark. So we made a decision that we would only deliver to 12 months and then we would send a text message with a link to the 15 and 18 month session information at that age appropriate time. The fact that we've been able to change the program, that's actually been a real positive for us and I think that's where other programs fall down [regional LGA]
Complexity	When I [dietitian] got notice about the training I contacted [the MCHN manager].. she already knew and had advocated and achieved the funding for three MCHNs to go - because clearly there's not much point in doing it [INFANT training] if they [MCHNs] are not on-board. So that was a significant driver [to do the training with them] [metro LGA]	To bring in another program, it had to mesh in with what we already had [metro LGA]<br>For our organisation, it was about looking for places to run the program… close to where [vulnerable] families were living.... and then organising some venues in those areas. Then it was about all the admin side of things, I suppose, which was huge [regional LGA]	I think the challenge for us is being in a regional/rural area you actually have to have enough births per year to support it being a sustainable program, and I think we only just have enough here. So it's getting the babies the right age at the right time and a core group of them, we could spend an awful amount of time getting that sorted if we [Community Health] didn't go through MCH services [regional LGA]

Design quality and packaging	I found it [INFANT training] useful. I really loved all the resources and I've used those. I've looked at the website and I think that's great as well with lots of practical ideas for parents and also for facilitators [metro LGA]<br>It was enough to get you started. I thought yes good resources, all the information is there and looking at it now there are probably a few things that I would like to see added in .....such as a focus on body image and "health at every size" [metro LGA]<br>I felt like the training was great...teaching how to do group skills and how to get parents talking to each other .. to have them empowering each other [metro LGA]	It [INFANT training] was very informative, because it went step-by-step and what people would be expected to do.. [metro LGA]<br>It definitely improved the handouts that they gave out to parents [metro LGA]	It [INFANT training] gave me a better background on the program...it was good to just see in detail what each session should look like. It was more just about how to actually run the program really. I think the only thing that I missed was how to evaluate if the program was successful or not, so maybe a bit more on evaluation would have been handy [regional LGA]<br>I don't think it's new information to us because this is our bread and butter. It's more how it's delivered, and the routine of the delivery - like with the video clips and questions that we're asking. The training was fantastic and I got so much information out of it, yeah I enjoyed it [metro LGA]<br>The only hiccups would be with the videos not working... because we don't have WiFi at all the centres {can't use the website link} [metro LGA]
Cost	Even knowing that well it has been done and there are organisations that have done it without funding and this is how they went about it [metro LGA]<br>I think [INFANT] it's a great program, and I would love to be able to run the whole thing. It's always confined, usually, around finance and staffing ... there's lots of competition for the available resources being staff and money [metro LGA]	Healthy Together gave us the funding to run it for 18 months. [but] they wouldn't pay for interpreters. We had to get our English clientele, which was hard for us [metro LGA]<br>Our senior management became aware of the [Healthy Together] funding and they alerted us to the fact that this [INFANT] program was up and coming [regional LGA]	Getting people in, that's Maternal and Child Health's responsibility. Then we [Community Health] have admin support that sends out all the text messages, books all the rooms for the groups, and then the dieticians just come in and deliver. That's another big positive for us… that the program does allow us to share that out [regional LGA]<br>Our council actually gives us extra funding so we can offer the [INFANT] program. So the reason we can run so many [sessions] and offer so much support is because we have the funding to do it [metro LGA]
**INNER SETTING**			
Organisational culture	[The MCHN group] was talking about offering [INFANT] as an add on for people to opt into.... so the risk was that you would have the most highly motivated people coming and those who you wanted to reach wouldn't access it. So, it really was pretty disappointing and it left me with the feeling that it wasn't enriching what we were going to be trying to do anyway.....so to be honest I lacked a champion there really [metro LGA]	To me it was a program that you could implement in other areas other than just maternal and child health [metro LGA]<br>The ability to do it properly and to reach those harder to reach, and to keep them coming and to get all the admin done, and all that really needs a designated team, I think. We ran the INFANT Program as part of what we were already doing. We were a bit caught between what we were also doing with our other hats on. Even though there was nice cross-pollination, [the program] needed to be established it in its own right … I think in the end there was a bit of uncertainty about whether or not MCH and us were doing similar stuff [regional LGA]	Maternal and Child Health is delivered from council and the dieticians work at the Community Health Services. ... so it doesn't fit anywhere nicely in our organisation, but I think you sort of can make it fit with the DHHS funding. So we put it under the Community Health which works for us having the dieticians deliver it [regional LGA]<br>From an administrative perspective, we've just adopted it [INFANT] as our own and we apply the same focus and method in facilitating it and coordinating it [metro LGA]
Organisational climate (tension for change, compatibility, relative priority)	I think the timing wasn't really right for us in the end. We'd merged with a much bigger organisation and a new CEO. [metro LGA]<br>I had MCH nurses saying mums aren't meeting after the child's turned 10 months. So, they were sort of scratching their heads saying we can't roll it out in this model [metro LGA]	What it did was give us more time to actually physically demonstrate it - provide toys for them to get on the floor and appropriate ways of interacting through age groups. It just gave you more space to do those things that you would only normally have a normal half hour to do in a consult [metro LGA]<br>Because we had like 10 programs going at a time, to have staff to run another program or an extra session was really, is a real strain …..Too many other programs [metro LGA]	We [Health Together LGA team] made a decision right back at the start that if it was going to keep going beyond the funding time, we needed to embed it in the services that we already have [regional LGA]<br>We're using the INFANT as a reason to stay together for participants of the first time {mothers} playgroup. We find that most people who come to the 3month INFANT {session} are usually those participants that are flow on from that {first time mothers playgroup} [metro LGA]<br>What I've been really advocating for is the really early years intervention so 0-4years because I think in the past we've always focused on schoolkids. So yes, more and more we've been looking at establishing good habits early on so when they get to school that it's already there [regional LGA]

Organisational readiness (leadership engagement, available resources)	I didn't think that I'd have a hope of being able to coerce the council into setting it up as six sessions [metro LGA]<br>It really was a capacity issue - if you were going to introduce INFANT, it would be more staffing and adding on extra groups [metro LGA]<br>We (dietitians) just couldn't do it ourselves. If someone said "here you go, two days a week, off you go" we could have done it. But we just didn't have that capacity there. We just said, "we just can't do it because it takes too much time away from our delivery." If maybe we knew that MCH nurses were interested or if some of them had been trained…. It's always a resource issue in community health [metro LGA]<br>The (MCHN) manager was trying to progress it by sort of saying well we could provide the administrative support and we could provide a venue but pretty much we'd need you (dietitian) to run it. I was sort of saying well I actually don't have the capacity to run it [metro LGA]	MCH nurses play a key part in that sort of education for new mums, around nutrition and play. So we wanted to make sure that we weren't treading on their toes, or doubling up or doing what it was that they were already doing, and if so, how could we do that well together? It was useful to make a strong connection with them because they were instrumental almost all of the referrals [regional LGA]<br>Basically we were told it came down to {funding} rate capping.<br>I had to plead to run the last [INFANT] program due to staffing numbers and the retention rate of the families…. we didn't have the staff to do it, there wasn't the staff there to back fill. I hate to say it, the bottom line is funding. INFANT isn't the only program that we've run that when the funding stopped it stopped [metro LGA]<br>We incorporated it [INFANT] into the roles that we already had but it certainly stretched us that's for sure. The program's not running anymore, because the funding concluded [regional LGA]<br>It's difficult to justify running a group for one or two people and paying someone, even at a low rate, for two to three hours. It's really not a group if there's only one or two people there. [metro LGA]<br>The biggest issue was always resources. It was the ability to be able to be take a room to hire, to run a group or to get enough hours to run the extra sessions [metro LGA]	Management were very supportive and encouraged us to attend {the training}. So, myself and another dietician, as well as the maternal child health nurse, attended that training [regional LGA]<br>When the training was going around my bosses signed me up because they thought I'd be good at it and I'd enjoy it and I did [metro LGA]<br>Before I (dietitian) went down to the training I went around to our maternal and child health nurses and consulted with them first so I knew that I could pretty much just implement it as soon as I got back. I had a couple of meetings with them before the training and then after the training it was more of a formality so I could give them a bit more detail on what I was going to cover in each session. I got them all on board about three months before I did the training [regional LGA]<br>I think the other thing that's helped is I have health promotion hours, so I do three days a week working with 0 to 12year olds so I've just been able to incorporate it [INFANT Program] into my role whereas I think a lot of other dieticians or maternal and child health nurses don't have that flexibility there [regional LGA]<br>If we didn't coordinate it through Child and Maternal Health, it would take us just too much administrative time to get those groups together so we wouldn't be able to run it [regional LGA]<br>We always had good intentions of implementing the INFANT Program, it was just about how we were going to do this with our staffing levels [regional LGA]<br>Our council actually gives us extra funding so we can offer these programs. So the reason we can run so many INFANT programs and offer so much support is because we have the funding to do it [metro LGA]
**OUTER SETTING**			
Patient needs and resources	I think that people haven't got any idea what it is or the benefit around it. I guess maybe some advertising or something [metro LGA]<br>We need to know about how to implement it with current technology... introducing the electronic form of it. Because most people have got smartphones [metro LGA	After 18 months you have that natural drop-off of people going back to work. I think that people were forgetting because there was distance between the age groups. We tried to bring it closer together so they actually got to come to the group halfway between the key age visits. We sent them notices to remind them [metro LGA]<br>A challenge was reaching vulnerable mums, and a lot of that was around transport. So they weren't always able to get to where the nearest session was, and there were times when we needed interpreters and we didn't have the dollars or the manpower to do that [regional LGA}<br>I think by the time parents got to 12 months they were feeling like they kind of had got over that hurdle of introducing food [regional LGA]<br>We have one first parents group that is Korean. Our parent support worker is also a bi-cultural worker in another organisation, so she does two [INFANT Programs], so language wise was fine, because she spoke the language [metro LGA]	I think with our community we've got a lot of disadvantaged families and I think a big thing is the hospital's not easy to get to if you don't have a car whereas [organisation's venue] is in the middle of town.... it's funded by the community and runs all these parent groups and the parents seem to have a good buy-in there. So yes, parents in our town are more likely to go to something run by that organisation rather than the hospital [regional LGA]<br>The way the program is structured, with mums sharing their progress, provides a good way of increasing confidence, because you can reinforce what mums are doing well [regional LGA]<br>I find as a maternal and child health nurse introducing solids and things like that are quite confronting for families and that extra support and that extra time is really the strength of it [INFANT]. It actually complements the first time {mothers} playgroup because it acts as that flow on, and it further supports the parents right up to the 12 month term. I believe

			and feel that it covers the parents' needs for that age group, and it's all relevant [metro LGA]
Cosmopolitanism (networked with other organisations)	Maybe some ongoing support or something that we could go to and say "this is our issue" and then someone says "you could approach it this way or this organisation actually did that." So some sort of contact point that's interested in what we do locally. Yes, probably just talking about it with others [metro LGA]<br>There are organisations that you can work with in the community to particularly target the intervention to those vulnerable groups [metro LGA]	The INFANT [Deakin research] team would send me emails with "If there are any questions". I never needed to use that resource, but I knew that it was there [metro LGA]<br>It would have been useful to have someone you could call to talk through some of the issues or the challenges ..wanting to make sure that we're on track with that. I think [the coordinator] might have had some contact with other LGAs throughout the state. To just keep in touch with what they were doing and when things came up [regional LGA]	Another Healthy Together LGA invited our organisation to attend a [INFANT] training day with them. My manager had a few conversations with them, who was very supportive of the [INFANT} program. So, that positive feedback from another organisation that was already running the program, was favourable for running it here as well [regional LGA]<br>I think the support from Deakin [INFANT research team], particularly in the early stages was really good [regional LGA]<br>Probably just having some ongoing support following the training, maybe just a discussion forum, to just see how others are approaching things [regional LGA]
Peer pressure (competing with other organisations)	I worked at one of the pilot program health centres, so I was aware that it [INFANT] was going on [metro LGA]	The team from Healthy Together [in local council] must have approached them [MCHN services] {to implement INFANT} [metro LGA]<br>We sort of ran it in-house and weren't really involved with any other organisations [metro LGA]	That positive feedback from another organisation that was already running the program, was favourable for running it here as well [regional LGA]
External policy and incentives	I'm wondering whether it could be something that you coordinate with the Department of Education and Training and the MAV...whether it could be something that's discussed with them, around getting the program more well known in the maternal and child health circle [metro LGA]<br>Ideally if you were rolling out INFANT and then those families then went to kinder {childcare} had supporting policy and programs you've got more chance of sustainability [metro LGA]	It would be lovely to just be able to do it in a fully funded, dedicated way, as the INFANT Program. You know, through state or federal funding, so it's in the same way that other services are provided. So then you can dedicate, you know, staff and things to it [regional LGA]	Department of Health need to do what they did with Healthy Together and give a suite of healthy living programs to choose from, and decide what the policy direction is with Integrated Health Promotion funding. If they're focusing on a life course approach, they should then be recommending some evidence-based programs and approaches... everyone's doing something sort of different, and interestingly, INFANT was one of the only ones that sort of got traction from Healthy Together...and they then need to think about the training requirements that go along with that and support that to happen, you know, that they run a number of INFANT facilitator trainings that they pay for, and then networking people together that are delivering those programs. That's where you learn a lot of stuff about actual translation of the research into practice and how it looks in different areas is by talking to other people that have done it [regional LGA]
**IMPLEMENTATION PROCESS**			
Planning	We run six sessions for new mums {new parent's group} - the sixth session is around food, nutrition, health, introducing solids. The babies, then, are generally around four to five months old, so, that's the session that I worked around the INFANT program [metro LGA]<br>So we were thinking we would work up a project that would include INFANT … We thought that fits in really nicely as one of the strategies that we did [metro LGA]	What we struck up was a relationship with them [MCHNs], whereby they would, when mums were finishing their six weeks of mother and baby classes, we would then turn up at their fifth or sixth session, to talk about the [INFANT] program. So that actually worked really well [regional LGA]<br>Invitations were given to families at the first time parent's group that was run by MCH. But the program was then run by early childhood workers or family support workers [metro LGA]<br>The groups were actually run by a maternal and child health nurse and a parent support worker. It worked well because parent support workers are able to give practical advice...the nurses could give more depth (information [metro LGA]	The first session of INFANT is the last formal session of the maternal and child health group {the new parent's group} and we deliver that at the Maternal and Child Health Centre. We schedule them all - if you're a 3month group we schedule your six, nine, 12month appointments and we do that for all the groups that run in the year. So we do it right from the outset. If they want to opt out and they don't want to receive the {reminder} texts anymore, they just reply Stop and we don't send them the texts anymore [regional LGA]<br>We had discussions around how we were going to coordinate the program and recruit and communicate with the participants [regional LGA]<br>We invite them [mums] to join at a first time {mothers} playgroup, and its promoted at the 2, 4 and 8 weeks {KAS} visits... and of course, the nurses that are home visting will also promote. All the people that book into our programs will get a

			text reminder on the day or a couple of days before. We've got a series of scripted prompts. When you receive it on your mobile, there are three links - the date is a link to your calendar, the address links to Maps on your phone, and also the website link so that it takes them straight to the brochure that's on our [LGA] website [metro LGA]
Engaging (opinion leaders, champions, formally appointed implementers, external change agents)	Maybe peer trainers - community members who have been trained in INFANT. Maybe if a group of people in a local area were trained, they could go and chat about it to their friends. It could be just run as a little - almost like a playgroup type thing. Yes, sort of community champions I guess or volunteers [metro LGA]	We did think about ways that we could incorporate it into our supported playgroup program … the difficulty with that is, if you've got children from 0 to 5years coming {to the same session} [regional LGA]<br>We met with councils who were running it with dieticians [but] that makes it a very expensive program [metro LGA]	Management has always been on board [regional LGA]<br>The [LGA] has really embraced it. Our council actually gives us extra funding so we can offer these programs. [The LGA] is very much geared towards children and healthy starts for them. [metro LGA]<br>I feel quite strongly that it's really a dietician's gig. I think a lot of the curly questions that get asked, like I am a trained facilitator, but there is no way I can give that same depth of information as what the dieticians can. That's one thing. I think parents actually value having the dietetics input too. There's a bit of professional prestige I think around it for us too. [regional LGA]<br>Mothers are currently recruited via the MCH program, but it would be great to get mums via midwifery [regional LGA]<br>The parent support service has started an antenatal group that will be running for high risk mums. So that's something that we definitely need to hook into....to integrate INFANT and the dietetic service with that group [regional LGA]
Executing	n/a	We didn't do the INFANT Program at the ages that were set out. We tried to bring it [sessions] closer together so they actually got to come to the group halfway between the key age {KAS} visits. [metro LGA]<br>Around six months was the most heavily attended sessions, because it was all that stuff around introducing solids. I think because the sessions were designed to pre-empt a stage ahead, I think often they had information that they needed by the time it got to 12 months. We had to be a little bit creative about how we got them to keep coming. We had to do lots of phone calls and lots of texting and lots of emailing. We trialled getting a coffee van to come along.... First Aid was focused in on home safety, choking and burns.... and CPR. So we kind of just picked some main areas that seemed really relevant to that time, to use as a bit of a way of getting families in and that worked well [regional LGA]<br>The drop off was huge ... so we decided to run the first three or four sessions, and then put the next two {15 and 18months} together. The length of time between each session....I think that impacts on uptake. It would be ideal if they were closer together, but then, that misses the whole age messaging completely [metro LGA]<br>What we've continued to do is the 3 and 6 months [INFANT sessions] as part of the first time parent's group, but we haven't continued with the others [INFANT sessions] ... and with the group dynamic, we were happy with that [metro LGA]	I think it's been set out quite good. It's been very easy to pick up and run. It has taken a little bit of planning just because a lot of our key groups they've got a range of ages [regional LGA]<br>I think in the early stages the scheduling was quite difficult, because it does need to be at age specific times, and then juggling that with the dieticians' diaries was quite difficult originally, but we've come up with a reasonably good scheduling system now and instructions for how to schedule it so the sessions end up at the right time and the dieticians don't end up overloaded. I would say share the load if you can between a number of different people and a number of organisations if that is possible [regional LGA]<br>Our numbers weren't very good, and that was, perhaps, in part, because we were clashing with the baby rhyme time at the local library. So we redid the program to offer the four sessions - 3, 6, 9 and 12months – across the month… so mums are aware that if they miss the session that their group was scheduled in to, they could attend one the month before or the month after, it didn't really matter. A Well Women's Nurse attends the 3month session and one of the physios will come, generally, only for ten or 15 minutes, just to see how the bubs are progressing in terms of their developmental milestones with sitting and rolling. Mums can ask questions at that time.<br>A speech pathologist is coming to the 9month session. We thought that was a good time when, hopefully, bubs have made some progression with texture and drinking from cups. But, if they haven't, then, the speech pathologist can give some

			pointers or, perhaps, arrange individual consults if need be. [regional LGA]<br>We're running 3, 6, 9 and 12-18 month sessions. So the 12, 15 and 18month sessions are combined.....attendance for the older children around the 12 to 18 month age frame tends to taper off [metro LGA]
Reflecting and evaluating	n/a	There were requirements set around evaluation, and the Council also sets requirements around any groups that we run. We had to report back to Healthy Together and collect evaluations from all the participants, which was at each session ....we were meant to have a discussion on the value of the [HTV] program but it never occurred [metro LGA]<br>The [HTV] targets were actually really high. We had 300 families that went through the INFANT program, and I think the target was actually higher than that .... a little bit too high to be honest for the amount of [HTV] dollars that we had. So it was accepted that we did the best we could, given what we had [regional LGA]<br>We did a base survey and then we did a follow up survey completed by families... age group, country of birth, language, ethnicity, understanding of health, nutrition, child's diet, use of other council programs. It's been hard getting evaluations back from families [metro LGA]<br>We send out forms every six months, twice a year, are you happy with...? was there anything else you needed? what as your facilitator like? Then we've also done some sessions just, someone going into a group and just asking a whole range of questions .. about the venue, how else to get the information... We've also done, last year, about people who didn't go. Is there a reason, is there some other way we could do it? A lot of them it's just, we've got a social network, we didn't need it [metro LGA]	At the moment we're relying on Department of Health measures .. they're only from 4year, they don't focus on the 0 to 4year age group. I'd like some tools to use to maybe pre and post evaluate [regional LGA]<br>The other question would be around what are we supposed to be evaluating? What other questions are we supposed to be asking? Is it around more competence, or how many mums we're getting to turn up, or is there something else…? But, also, I guess, if we chose to evaluate the INFANT program a bit more formally within the organisation, we could write it up as a more formal report that would go to the CQI committee [regional LGA]<br>I think the other thing that we might chat about is following up on the DNAs {do not attends} and also following up on those that have attended a session but then don't continue on with the subsequent sessions [of INFANT] [metro LGA]
**FACILITATOR CHARACTERISTICS**			
Knowledge and belief about intervention	I guess what has come out of that (attending INFANT training) is a real passion for doing a bit more around early childhood ...it's sort of in me now that the early years are the most important [metro LGA]<br>I felt like the training was great... [But] I already know how to run groups, I know how to get parents talking with each other, I've done that thousands of times. What would help us is to know how better to deliver it in our setting [metro LGA]	The philosophies of the INFANT program were important to us [program facilitators]. We thought the program was easy to deliver [metro LGA]<br>The training really just gave the staff more confidence … reinforced what they already knew, it reinforced what they were teaching [metro LGA]	I don't think it's new information to us because this is our bread and butter. It's more how it's delivered [metro LGA]<br>We (MCHNs) really like the program and what it stands for [metro LGA]<br>I was actually challenged by the training, because there was a strong focus on facilitating the program. In the training, the focus was more on just active listening and not so much information delivery. I probably do like to take a little bit more control, rather than just be led by the participants. That was a little bit challenging for me [regional LGA]
